# Physico-chemical studies of resveratrol, methyl-jasmonate and cyclodextrin interactions: an approach to resveratrol bioproduction optimization†

**DOI:** 10.1039/c7ra11619e

**Published:** 2018-01-04

**Authors:** E. Oliva, D. Mathiron, E. Bertaut, D. Landy, D. Cailleu, S. Pilard, C. Clément, E. Courot, V. Bonnet, F. Djedaïni-Pilard

**Affiliations:** Laboratoire de Glycochimie des Antimicrobiens et des Agroressources, LG2A UMR 7378 CNRS, Université de Picardie Jules Verne 33 rue Saint-Leu 80039 Amiens France florence.pilard@u-picardie.fr; Plateforme-analytique, Université de Picardie Jules Verne 33 rue Saint-Leu 80039 Amiens France; Unité de Chimie Environnementale et Interactions sur le Vivant (UCEIV, EA 4492), ULCO F-59140 Dunkerque France; Unité de Recherche Vignes et Vins de Champagne, (URVVC, EA 4707), Université de Reims Champagne-Ardenne, UFR Sciences BP 1039, Moulin de la Housse 51687 Reims France; SFR Condorcet “Agrosciences Environnement et Développement Durable” FR CNRS 3417, UFR Sciences Exactes et Naturelles BP 1039 Moulin de la Housse – Bâtiment 18 51687 Reims Cedex 02 France

## Abstract

*trans*-Resveratrol (RSV) is a natural phenolic molecule of the stilbene family known for its anti-oxidant properties in the field of nutraceuticals and cosmetics. Its production by grapevine cell suspensions is induced by the addition to the culture medium of elicitor compounds, methyl jasmonate (MeJA) and cyclodextrins (CDs). Physico-chemical studies were performed to understand the mechanism of action of CDs on this bioproduction of RSV. Inclusion complexes of RSV in CDs were first observed and then interactions with MeJA were identified using various analytical techniques such as UV and nuclear magnetic resonance (NMR) spectroscopies, mass spectrometry (MS) and isothermal titration calorimetry (ITC).

## Abstract 2

The role of cyclodextrins in the bioproduction of resveratrol in the presence of methyl-jasmonate is rationalized, leading to solutions for the improvement of bioproduction.

## Graphical abstract

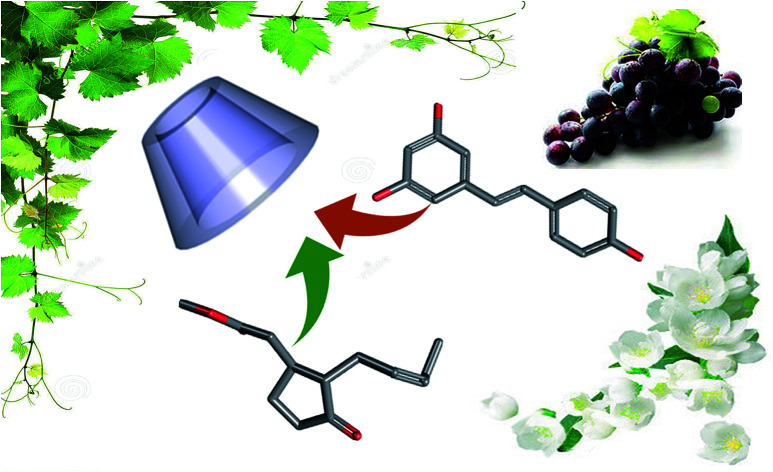

## Introduction

**Fig. 1:**
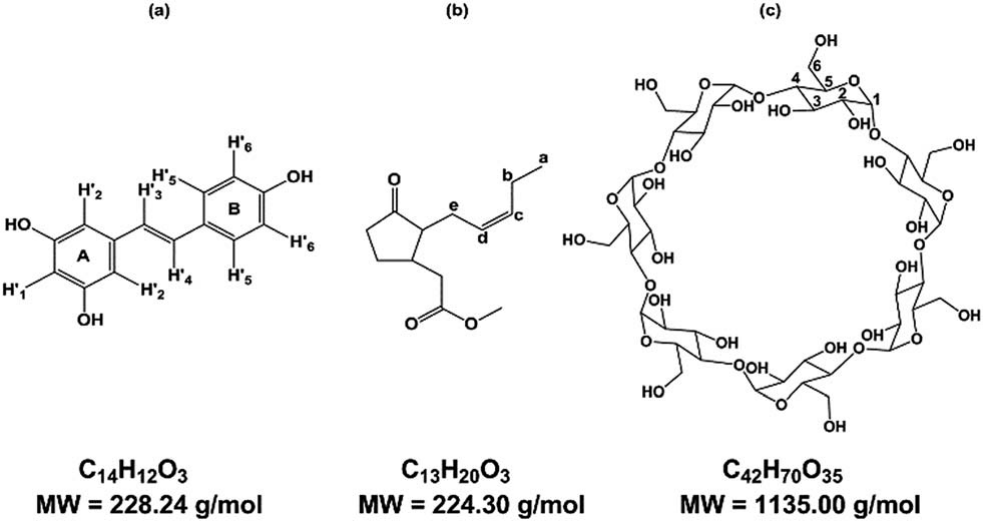
Structure of (a) *trans*-resveratrol (RSV), (b) methyl jasmonate (MeJA), (c) β-cyclodextrin (β-CD).

Known to be involved in plant defense mechanisms, *trans*-resveratrol or 3,5-4′ trihydroxy-*trans*-stilbene, RSV, (Fig. 1a) and its derivatives, so-called viniferins, have been shown to protect grapevine species or cultivars against fungi such as downy and powdery mildew.^1,2^ Moreover, its extension of the lifespan of different organisms has been described and RSV is renowned as the major agent responsible for the *French Paradox*, based on the notion that daily consumption of red wine is good for human health. RSV acts against cardiovascular disease, cancer, diabetes, obesity, inflammation and neurodegenerative diseases.^3,4^

Due to its properties, RSV is in high demand, and one way to meet the demand is through extraction from plant natural sources as roots of *Fallopia japonica* (nutraceutic use)^5^ or grape canes (cosmetic use).^6^ However, these extraction processes are not very sustainable and the bioproduction of RSV has been developed using recombinant microorganisms and plant cell cultures. As the use of bacteria or yeasts needs genetic transformation, grapevine cell cultures remain a good alternative to obtain RSV and various stilbenoids.^7,8^ Plants have developed complex strategies for protection against diseases and, more widely, stress. The study of such mechanisms led to the identification of agents of plant cell perception, molecules named elicitors that could stimulate the defense responses through signal molecules such as chitosan, jasmonate or methyl jasmonate (MeJA) (Fig. 1b).^9–11^ These compounds stimulate the synthesis of PR proteins or of small molecules of the secondary metabolism involved in the defense, so called phytoalexins, such as RSV. MeJA is currently used as an elicitor, especially in grapevine cell cultures where it can induce or enhance the quantity of stilbenes.^7^ Although high RSV production has been obtained through the use of MeJA, the excretion of the molecule is followed by a rapid decrease in RSV quantity, due to its degradation in the culture medium, especially by oxidation.^8^ CDs have been tested for their ability to complex RSV in its hydrophobic cavity to overcome the poor solubility and the oxidation damage of this polyphenol compound.^12^

CDs are starch-derived cyclic oligosaccharides composed of α-1,4-linked d-glucopyranoside units. The most common ones are α-, β- and γ-CD, with 6, 7 and 8 units, respectively (Fig. 1c). CDs have a truncated cone-like three-dimensional structure. The hydroxyl groups are oriented towards the outside of the cone, which gives CDs an overall hydrophilic character.

The protons H3 and H5 and the inter-glycosidic oxygens are oriented towards the interior of the cavity, conferring a hydrophobic character on the cavity. The other protons, H1, H2, H4, and H6, are directed towards the outside. This cavity thus has supramolecular inclusion properties in aqueous solution.^13,14^ Some chemical derivatives of CDs, such as DIMEB (2,6-di-*O*-methyl-β-CD) and RAMEB (randomly methylated-β-CD), are also available and exhibit inclusion properties despite significant differences in hydrophobicity, solubility, and conformational flexibility.^15^

The results of RSV bioproduction is clearly influenced by the type of CDs used. It is now well known that a high concentration of RAMEB (50 mM, 65.5 g L^−1^) could lead to several g L^−1^ of RSV in the medium.^16^ Our *Vitis labrusca* cell line is not able to produce RSV without elicitation^17^ but β-CD (15 g L^−1^) and RAMEB (65.5 g L^−1^) are able to induce its biosynthesis, leading to 32 mg L^−1^ and 262 mg L^−1^ of RSV with β-CD and RAMEB, respectively. Moreover, both MeJA (0.8 mM) and CDs (15 g L^−1^) elicitation were tested, yielding 170 mg L^−1^ and 274 mg L^−1^ of RSV in the medium with RAMEB and β-CD, respectively. Other groups have already described this effect.^18,19^ The application of these results in a 14 L bioreactor led to a lower RSV production of 72 mg L^−1^ with β-CD (15 g L^−1^) and MeJA (0.8 mM)^17^ confirming that a scaled-up optimization is necessary. CDs are able to activate the genes encoding enzymes from the stilbene pathway and this effect could be enhanced by MeJA addition.^20^ RSV biosynthesis is highly induced by the presence of these elicitors but RSV is clearly oxidized in the medium of culture if no CDs are present. The addition of CDs has an inhibitory effect on resveratrol oxidation,^21^ maintaining its concentration at a high level in the medium of culture. A few articles showed that RSV is included in the cavity of the α-, β-, γ-CD, hdroxy-propyl-β-cyclodextrin (HP-β-CD), DIMEB,^22^ carboxymethyl-β-CD, acetyl-β-CD and maltosyl-β-CD.^23^ Some structural data were given for these complexes too.^24,25^ Unfortunately, the mechanism of action of CDs and MeJA simultaneously on the RSV production process remains uncertain and complex. Nevertheless, it is clear that if CD/RSV and CD/MeJA complexes are formed in solution, many other species may be present in the medium at the same time, such as free RSV, CD and MeJA, binary RSV/MeJA or even ternary CD/RSV/MeJA complexes. The ratio of each species is dependent on the value of the different association constants.

In the present work, the aim was a deeper understanding of the mechanisms involved. The strategy consisted of first considering both CD/RSV and CD/MeJA interactions independently and then evaluating the case in which the three compounds could interact with each other. To probe these interactions, physico-chemical studies were undertaken by UV, MS, NMR and ITC. First, CD/RSV interactions were investigated using phase solubility diagrams, which revealed that RAMEB and β-CD were the best to solubilize RSV in water. This is in agreement with biological results and was confirmed by MS studies. A complete structural characterization of the RAMEB and β-CD/RSV complexes was performed by NMR and their stoichiometry, association constant values and thermodynamic parameters were obtained using ITC. Secondly, complexes with MeJA were rapidly screened by MS to check that RAMEB and β-CD gave the best results. Thereafter, the study focused on the characterization of the RAMEB/MeJA and β-CD/MeJA complexes by their solubility diagram, their structural analysis by NMR and the determination of their thermodynamic parameters using ITC. The interactions between the three components were also studied by ITC and NMR experiments such as ^1^H DOSY and T-ROESY. Finally, RSV chemical stability, under UV exposure, alone and in the presence of CDs and MeJA was investigated by LC/UV/HRMS. In agreement with the physico-chemical studies it was demonstrated that RAMEB and β-CD protect RSV from degradation.

## Results and discussion

### Study of CD/RSV interactions

First, we studied the interactions between various CDs used to elicit grapevine cell cultures and RSV to compare our results with previous data in the literature.

### Solubility studies

In the first approach, to support the choice of β-CD and RAMEB, their solubilizing effect on RSV in water compared to other natural CDs (α-CD, γ-CD) was explored. To do so, phase solubility studies according to the Higuchi and Connors method^26^ were performed to investigate quantitatively the inclusion complex phenomena.

**Fig. 2:**
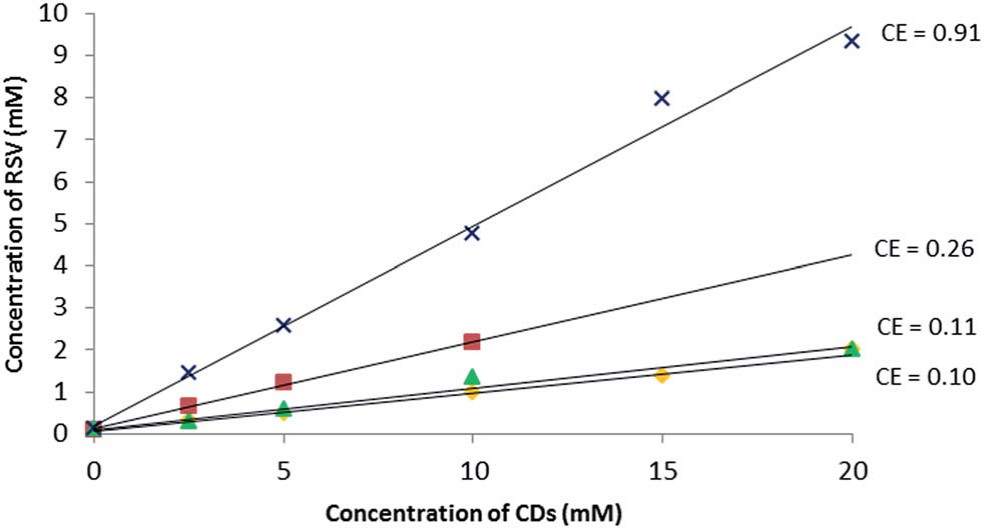
Phase solubility diagram of RSV in aqueous solution at 25 °C, pH between 6.7 and 7.8: α-CD (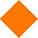), β-CD (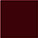), γ-CD (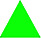) and RAMEB (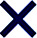) with dedicated CE values.

As depicted in Fig. 2, a linear increase in RSV solubility as a function of CD concentration was observed revealing A_L_ type profiles^27^ and suggesting the formation of a solution complex in all cases. Among the natural CDs, β-CD had the biggest solubilizing effect on RSV. Nevertheless, as the slope of the linear portion of the solubility curve was greater for RAMEB, this has a bigger RSV solubilizing effect than β-CD in water. Indeed, in the presence of 10 mM of β-CD and RAMEB, there was an increase in RSV solubility from 0.1 mM without CDs to 2 mM and 4 mM, respectively. There are very few phase solubility measurements in the literature: Bertacche *et al.* described an apparent solubility of RSV in the presence of RAMEB and γ-CD but the concentration ranges used were too weak (0 to 1.2 mM) to be significant.^22^ Conversely, Zhou *et al.* obtained similar results to ours with β-CD and HP-β-CD in the same range of concentrations.^28^

Moreover, it is often assumed that the linear trend of the phase solubility diagram indicates the formation of a 1 : 1 complex. For a guest molecule with limited aqueous solubility such as RSV (0.1 mM at saturation), it is well-known that the association constant value cannot be determined accurately with this method (Table SI†) and the notion of Complexation Efficiency (CE) was introduced by Loftsson *et al.* to compare the solubilizing effect of different CDs on a guest molecule.^29^ The highest CE value of 0.91 was found for RAMEB and the results can be summarized as follows:CE α-CD < CE γ-CD < CE β-CD < CE RAMEB (Fig. 2).

### MS studies

**Fig. 3:**
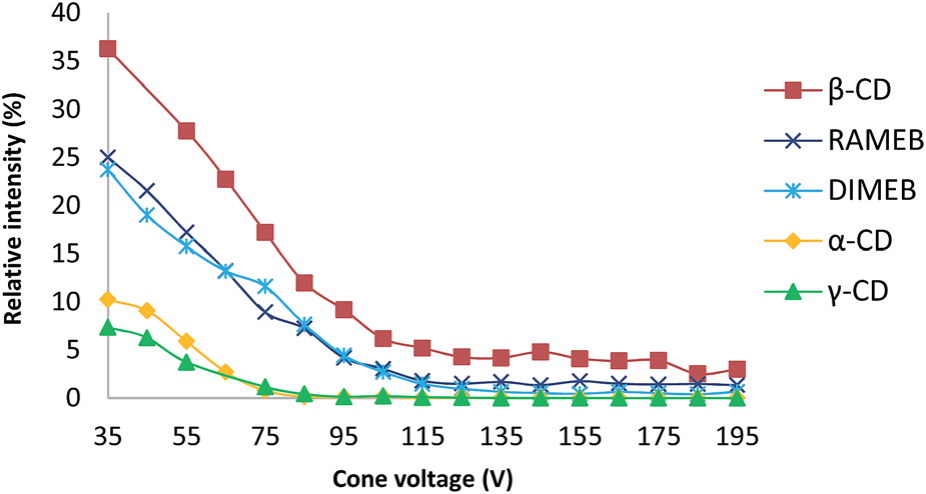
Plot of the relative intensity of each complex between RSV and different CDs *versus* cone voltage (for details, see the experimental part).

ESI ionization is a well-known soft ionization method that can transfer non-covalent complexes from the liquid to the gas phase by keeping them intact.^30^ To probe the stability of each CD/RSV complex, in source CID fragmentation experiments were performed, which consisted of applying increasing cone voltage values (35 to 195 V) and providing enough energy to dissociate the ions corresponding to these complexes (Fig. 3). The more stable the complex, the more energy must be applied. From these experiments (Fig. 3), it was found that complexes with β-CD and methylated CDs (RAMEB, DIMEB) were more abundant and more stable than those involving α-CD and γ-CD, which is in agreement with previous results obtained in phase solubility studies. In order to check unambiguously that the *m*/*z* of the ions corresponding to the sum of the masses of the CDs and RSV were really complexes and not just adducts formed in the electrospray source, maltoheptaose,^31^ a linear oligosaccharide homologue of β-CD was used and no signal corresponding to the association of maltoheptaose and RSV was observed (Fig. S1†). Consequently, the complexes observed with CDs and RSV by MS were very specific and certainly resulted from the formation of inclusion complexes.

### NMR studies

To confirm the formation of inclusion complexes between the tested CDs (α-CD, β-CD, γ-CD, DIMEB (RAMEB model for NMR)) and RSV, ^1^H NMR experiments were first carried out. As the rather low RSV solubility hindered reliable investigations by Job Plot and titration experiments (Fig. S2†), a comparison of ^1^H NMR spectra of CDs (4.4 mM in D_2_O) in the absence and presence of RSV at saturation was performed to highlight chemical shift variations on the H3 and H5 protons located inside the CD cavity. The addition of RSV to CDs led to upfield chemical shift variations on every CD proton, especially for H3, H5 and H6 (Table 1) revealing the formation of inclusion complexes between RSV and all the CDs.

**Table 1:** Chemical shift variations observed for each proton of CDs in the absence and presence of RSV (4.4 mM, D_2_O, 600 MHz at 300 K)

Proton	Δ*δ* 10^−3^ (ppm)			
	α-CD	**β-CD**	γ-CD	**DIMEB**
H1	4	8	4	55
H2	8	8	3	27
**H3**	19	**28**	7	**71**
H4	6	2	2	47
**H5**	ND^a^	**51**	ND^a^	**125**
**H6**	ND^a^	**30**	7	**89**

a ND: not determined due to overlapping signal.

It should be noted that the largest chemical shift variations were observed for β-CD and DIMEB with a noticeable shielding for H3, H5 and H6, suggesting a strong interaction with RSV. Therefore, β-CD and RAMEB were selected as the best candidates to use in our study on the basis of solubility studies, MS and ^1^H NMR results, in agreement with their biological effect of inducing high RSV production in grapevine cell cultures.

**Fig. 4:**
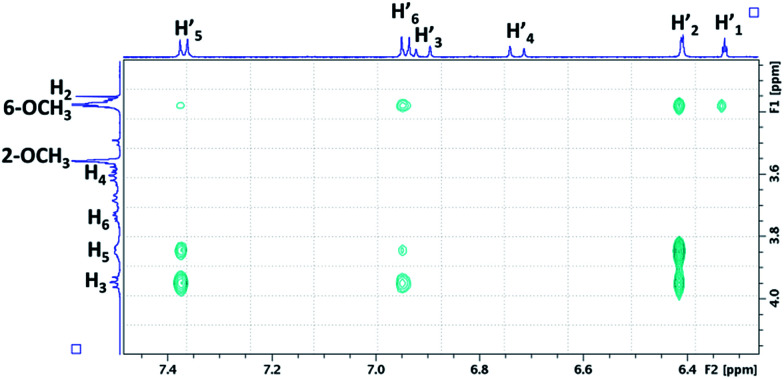
Partial contour plot of a T-ROESY experiment (spin lock: 300 ms, 22 dB) performed at 600 MHz on the DIMEB/RSV inclusion complex (4.6 mM of DIMEB with RSV at saturation in D_2_O). Vertical scale, CD area; horizontal scale, aromatic area of RSV.

We focus now on the characterization of β-CD/RSV and DIMEB/RSV inclusion complexes using T-ROESY experiments. As depicted in Fig. 4, the presence of cross-correlation peaks between aromatic protons of RSV and those from the DIMEB cavity fully supports the formation of an inclusion complex. Strong interactions between the H3 and H5 protons of DIMEB and protons of both RSV aromatic rings (mainly H-2′ of ring A and also weakly H-5′ and H-6′ of ring B) were observed. It should be noted that no interaction with H6 of DIMEB was observed whereas a correlation between the methoxy group in position 6 of DIMEB with all the RSV aromatic protons (H-1′, H-2′, H-5′, H-6′) was highlighted. Conversely to the 3D inclusion complex structures proposed by Lu *et al.*^32^ and Ishikawa *et al.*,^33^ no interaction with the ethylenic protons H-3′ and H-4′ in the central position of RSV was observed. As a 1 : 1 stoichiometry can be postulated on the basis of the RAMEB/RSV solubility study, these NMR observations probably indicate that DIMEB/RSV inclusion complex exists as two types of 1 : 1 complexes involving each aromatic moiety.

For the β-CD/RSV inclusion complex, similar cross-correlation peaks were found involving H3 and H5 of β-CD with the aromatic protons of RSV (data not shown). Based on MD simulations and T-ROESY experiments, Troche-Pesqueira *et al.*^34^ suggested that the preferential 1 : 1 inclusion complex should be that in which the aromatic ring A of RSV is oriented towards the narrow rim of β-CD. Conversely, Sapino *et al.*^35^ proposed a preferential inclusion by the B ring moiety with HP-β-CD by using silico design. Unfortunately, competing processes in solution such as aggregation phenomena of RSV, as already demonstrated by Lopez-Nicolàs & García-Carmona,^36^ precludes a definitive picture of the β-CD/RSV geometry. It should be noted that only one Job Plot study has been described in the literature between RSV and cyclodextrin derivatives, but it was performed in an equimolar MeOH/H_2_O solution. In these conditions, the aggregation phenomenon would probably be restricted.^37^

In addition to T-ROESY data, DOSY experiments also confirmed the interaction of RSV with β-CD and DIMEB *via* the formation of an inclusion complex due to changes in the diffusion coefficient *D* value. In fact, the diffusion coefficient is a parameter that can be linked to the translational motion of molecules in solution and whose value depends on the size of the object. The bigger the molecule is, the smaller the *D* value will be. For instance, RSV alone in D_2_O revealed a *D* value of 6.19 × 10^−10^ m^2^ s^−1^ whereas a value of 2.75 × 10^−10^ m^2^ s^−1^ was observed for β-CD alone. In the presence of β-CD, a decrease in the RSV *D* value was observed, reaching a value of 3.29 × 10^−10^ m^2^ s^−1^ very close to that of β-CD alone, revealing an interaction *via* the formation of a β-CD/RSV inclusion complex. The same phenomenon was observed with DIMEB with a decrease in the RSV *D* value from 6.19 × 10^−10^ m^2^ s^−1^ to 2.37 × 10^−10^ m^2^ s^−1^. On the other hand, it should be noted that CD *D* values were barely affected by the formation of CD/RSV inclusion complexes with Δ*D* of 0.01 and 0.2 × 10^−10^ m^2^ s^−1^ for β-CD and DIMEB, respectively. Because of the equilibrium occurring in solution between free RSV and its complexed form, RSV, which is the guest, will overall take the motion in solution of its host during the inclusion process. Therefore, DOSY experiments are a very useful tool to probe non-covalent interactions.

### ITC studies

**Fig. 5:**
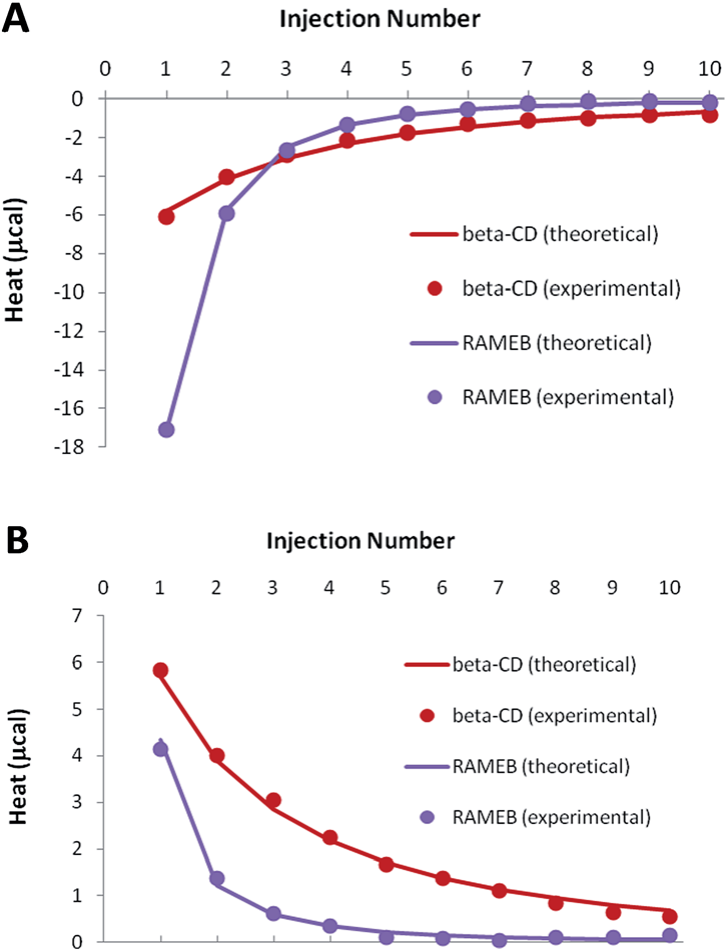
Experimental (dots) and theoretical (solid lines) ITC isotherms obtained for RSV complexation by β-CD and RAMEB, in titration (A) and release (B) experiments.

As demonstrated by our solubility studies, the RSV concentration must be lower than 0.1 mM in the absence of CD, in order to obtain homogeneous solutions. As a result, ITC titrations of RSV should be carried out with rather dilute solutions to avoid any unspecific heat due to aggregates. Due to such low concentrations, weak negative heats were recorded when RSV was titrated by CDs, especially in the case of β-CD (Fig. 5A).

In order to increase the accuracy of the thermodynamic characterization, additional release experiments were performed, by injecting the CD-RSV complex within the cell, initially filled with buffer (Fig. 5B). As this protocol induces in the cell a partial dissociation of the complex that was pre-formed in the syringe, positive heats were observed. Simultaneous treatment of titration and release experiments led to a very good fit between the experimental and theoretical heats when considering a 1 : 1 stoichiometry, with satisfactory accuracy of the thermodynamic parameters (Table 2). The affinity was significantly higher for RAMEB compared to β-CD, which is in agreement with their respective solubilizing ability. If the complexation is exothermic for both CDs, this increase in affinity is mainly due to a greater entropic contribution in the case of RAMEB. The increased hydrophobic cavity of the methylated oligosaccharide seems to be better suited to include RSV than the natural CD. ITC unambiguously confirmed the formation of 1 : 1 β-CD/RSV and RAMEB/RSV complexes, as previously underlined by our solubility studies. In addition, the use of the formation constants presented in Table 2 allowed a perfect reproduction of solubility data (Fig. S3†), thus confirming the reliability of affinities measured by ITC.

**Table 2:** Thermodynamic parameters obtained from ITC experiments for β-CD/RSV and RAMEB/RSV complexes

Stoichiometry	β-CD/RSV	RAMEB/RSV
	1 : 1	1 : 1
*K*	2415 ± 99 M^−1^	18 910 ± 930 M^−1^
Δ*H*	−3549 ± 817 cal mol^−1^	−3219 ± 264 cal mol^−1^
−*T*Δ*S*	−1061 ± 841 cal mol^−1^ K^−1^	−2609 ± 293 cal mol^−1^ K^−1^

For the β-CD/RSV complex, the association constant value of 2415 M^−1^ is in agreement with the literature, which reports values of 2205 M^−1^ by the fluorimetric method^19^ and 1922 M^−1^ by HPLC-UV.^38^

Nevertheless, the determination of the association constant value is method-dependent and overestimated values have been found using the enzymatic method (4220 M^−1^) and the solubility method (4438 M^−1^).^23^ In the case of methylated cyclodextrins (DIMEB or RAMEB), very few complexes have been described in the literature, with underestimated *K* values in the range of 2000 to 3000 M^−1^.^22,39^

The significant results are summarized in Table 3 and demonstrate unambiguously that RAMEB (or DIMEB) forms the strongest complex with RSV. The ratio between the association constant values obtained with RAMEB (18 910 M^−1^) and β-CD (2415 M^−1^) is 7.83. It should be noted that the RSV production from 32 mg L^−1^ to 262 mg L^−1^ with β-CD and RAMEB, respectively, gave a ratio of 8.19. We can conclude that the improvement in RSV production using RAMEB is mainly due to its enhanced solubility and perhaps its oxidation protection by formation of a very robust inclusion complex.

**Table 3:** Solubility of RSV, association constant (*K*) and variation in diffusion coefficients (Δ*D*) in the presence of CDs (4.6 mM in water at 300 K)

CD	RSV solubility (mM)	*K* (M^−1^)	Δ*D* (10^−10^ m^2^ s^−1^)
β-CD	1.09	2415	−2.9
RAMEB	2.36	18 910	−3.82

### Study of CD/MeJA interactions

Although the elicitation effects of MeJA and cyclodextrins have been extensively studied, only very few works have described the potential interactions between MeJA and CDs in solution. López-Nicolás *et al.* demonstrated by HPLC in a binary solvent mixture (MeOH/H_2_O 40 : 60) that the interactions of MeJA with β-CD and HP-CD were more efficient than with α-CD and γ-CD but the measured association constant values remained modest (40–60 M^−1^) compared to those obtained with RSV.^40^ Thus, the same experimental strategy applied previously was carried out with MeJA.

### MS studies

To study CD/MeJA interactions, the same MS methodology was applied as previously described using in source CID fragmentation experiments (Fig. S4†). Less obviously than in the case of CD/RSV complexes, complexes involving methylated CDs (DIMEB, RAMEB) and β-CD with MeJA were hardly more abundant and more stable than those involving other CDs. Since an *m*/*z* corresponding to the association of MeJA with maltoheptaose was observed unlike RSV, a weak contribution of the non-specific association of MeJA with the outside of CDs can be expected.

### Solubility studies

**Fig. 6:**
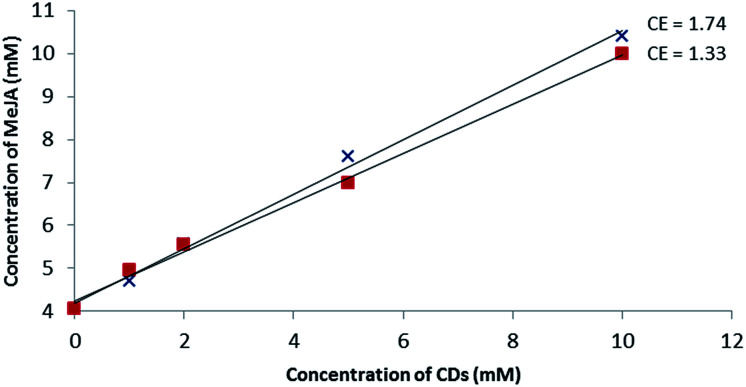
Phase solubility diagram of MeJA obtained by the ERETIC method in D_2_O solution at 25 °C: β-CD (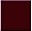) and RAMEB (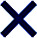).

Based on the MS studies, the solubilizing effect of β-CD and RAMEB on MeJA in water was explored. To our knowledge, no solubility study of MeJA in the presence of cyclodextrin has been described in the literature. Moreover, in the absence of an efficient chromophore, the phase solubility study by UV/Vis according to the Higuchi and Connors method failed and was performed using a dedicated ^1^H-NMR quantitative method called ERETIC.^41^ As depicted in Fig. 6, a linear increase in MeJA solubility as a function of CD concentration was observed revealing A_L_ type profiles, suggesting the formation of a solution complex in both cases. Since both slopes of the linear portion of the solubility curve are similar, it can be assumed that the association constant values are in the same range. With this method, the MeJA solubility at saturation in water was estimated at 4.1 mM and the solubilizing effect of CDs was found to be larger than with RSV with CE values of 1.33 and 1.74 for β-CD and RAMEB, respectively.

### ITC studies

**Fig. 7:**
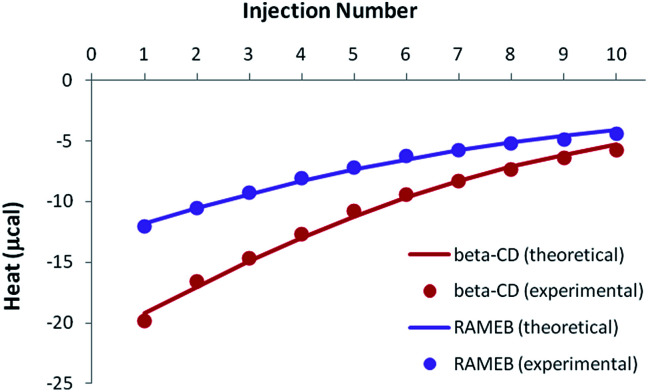
Experimental (dots) and theoretical (solid lines) ITC isotherms obtained for MeJA titrations with β-CD and RAMEB.

Because of the greater solubility of MeJA compared to RSV, satisfactory heats were observed when performing ITC titrations with β-CD or RAMEB (Fig. 7). Theoretical heats for a 1 : 1 inclusion complex were in very good agreement with the experimental signals, considering a 1 : 1 equilibrium.

Accurate thermodynamic parameters could thus be obtained (Table 4), by using only one kind of ITC protocol. In the case of β-CD, the inclusion of MeJA was characterized by an affinity analogous to that measured for RSV. In contrast, the formation constant observed for the RAMEB complexation of MeJA was one order of magnitude weaker than in the case of RSV. In addition, MeJA complexes were stabilized by rather similar enthalpic and entropic contributions, whichever CD was studied. Finally, as in the case of RSV, solubility data were nicely simulated by using the formation constants determined by ITC (Fig. S5†).

**Table 4:** Thermodynamic parameters obtained from ITC experiments for β-CD/MeJA and RAMEB/MeJA complexes

Stoichiometry	β-CD/MeJA	RAMEB/MeJA
	1 : 1	1 : 1
*K*	2270 ± 232 M^−1^	1280 ± 180 M^−1^
Δ*H*	−2079 ± 155 cal mol^−1^	−1734 ± 230 cal mol^−1^
−*T*Δ*S*	−2494 ± 216 cal mol^−1^ K^−1^	−2504 ± 314 cal mol^−1^ K^−1^

### NMR studies

**Fig. 8:**
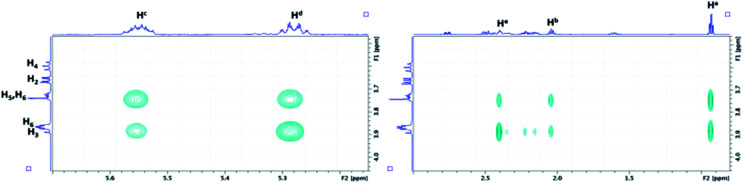
Partial contour plot of a T-ROESY experiment (spin lock: 300 ms, 22 dB) performed at 600 MHz on the β-CD/MeJA inclusion complex (4.6 mM of β-CD with MeJA at saturation in D_2_O). Vertical scale, CD area; horizontal scale, area of MeJA.

As depicted in Fig. 8, the characterization of β-CD/MeJA and DIMEB/MeJA inclusion complexes was performed using T-ROESY experiments. The presence of strong cross-correlation peaks between protons Ha, Hb, Hc, Hd and He, strictly located on the linear ethylenic chain of MeJA, and those from the CD cavity (H3, H5 and H6) fully supports the formation of a 1 : 1 inclusion complex.

The significant results obtained by NMR and ITC are summarized in Table 5. Similar solubilizing effects on MeJA are observed with β-CD and RAMEB, consistent with association constant values that are in a similar range and unlike those obtained with RSV. In addition to T-ROESY and ERETIC data, DOSY experiments also confirmed the interaction of MeJA with β-CD and DIMEB *via* the formation of inclusion complexes due to the decrease in the diffusion coefficient *D* value.

**Table 5:** Solubility of MeJA, association constant (*K*) and variation in diffusion coefficients (Δ*D*) in the presence of CDs (4.6 mM in water at 300 K)

CD	MeJA solubility (mM)	*K* (M^−1^)	Δ*D* (10^−10^ m^2^ s^−1^)
β-CD	6.88	2270	−2.75
RAMEB	7.10	1280	−2.17

### Study of CD/RSV/MeJA interactions

#### ITC studies

In order to investigate the complexing behavior of CDs in the presence of both RSV and MeJA, competitive release experiments were performed with ITC. The solubilizing ability of CDs towards RSV was again exploited in order to use consistent concentrations of both guests. A solution containing CD (5 mM) and RSV (0.6 mM in the presence of β-CD, 1 mM in the presence of RAMEB) was injected into the ITC cell, previously filled with a MeJA solution (0.5 mM). Following the hypothesis of competitive inclusions, such an experiment should induce the simultaneous dissociation of RSV complexes (endothermic signal) and the formation of MeJA complexes (exothermic signal).

If the previously determined thermodynamic parameters for the individual complexes and their respective concentrations are taken into account, inclusion complex formation should be greater than dissociation, in such a way that the overall signal should remain exothermic (the isotherm shape can be either monotonous or non-monotonous, depending on the studied CD).

**Fig. 9:**
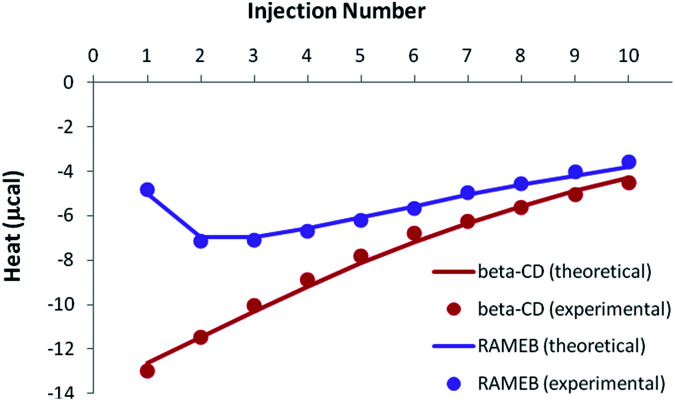
Experimental (dots) and theoretical (solid lines) ITC isotherms obtained for the competitive release of an RSV/CD solution within a MeJA solution.

In the case of the existence of a ternary complex, a third enthalpic contribution should be recorded upon formation of this additional inclusion compound. At this stage, it should be underlined that all isotherms were treated simultaneously, *i.e.* for both individual (see previous sections) and mixed studies of RSV and MeJA. As can be seen in Fig. 9, the competitive release isotherms were well reproduced when the model was only based on competitive phenomena, for both β-CD and RAMEB. It is clear that the thermodynamic parameters previously determined for individual complexes are sufficient to describe the heats recorded when RSV and MeJA are mixed together.

As a consequence, it can be concluded that ternary complexation did not take place.

#### NMR studies

**Fig. 10:**
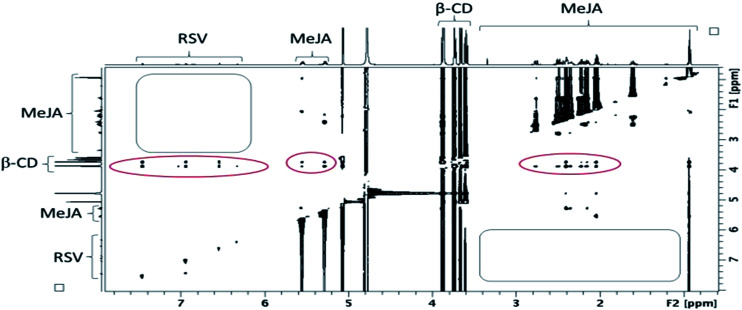
Contour plot of a T-ROESY experiment (spin lock: 300 ms, 22 dB) performed at 600 MHz on a β-CD/RSV/MeJA mixture (4.6 mM of β-CD with RSV and MeJA at saturation in D_2_O). Rectangles represent the absence of a correlation between RSV and MeJA and ovals the presence of correlations between CDs and RSV on one hand and MeJA on the other hand.

The previous hypothesis is completely confirmed by the T-ROESY experiment performed on the mixture of the three components (CD, RSV and MeJA) as displayed in Fig. 10.

In fact, the same correlations (inside ovals of Fig. 10) were observed between protons of CD (DIMEB or β-CD) and RSV or MeJA as those observed in the binary mixtures CD/RSV (Fig. 4) and CD/MeJA (Fig. 8) while no correlation (inside rectangles of Fig. 10) was highlighted between protons of RSV and MeJA as expected in the case of ternary complexation. It can be concluded that in the presence of the three compounds, both inclusion complexes (CD/RSV and CD/MeJA) co-exist in solution in amounts directly correlated to their association constant values.

**Fig. 11:**
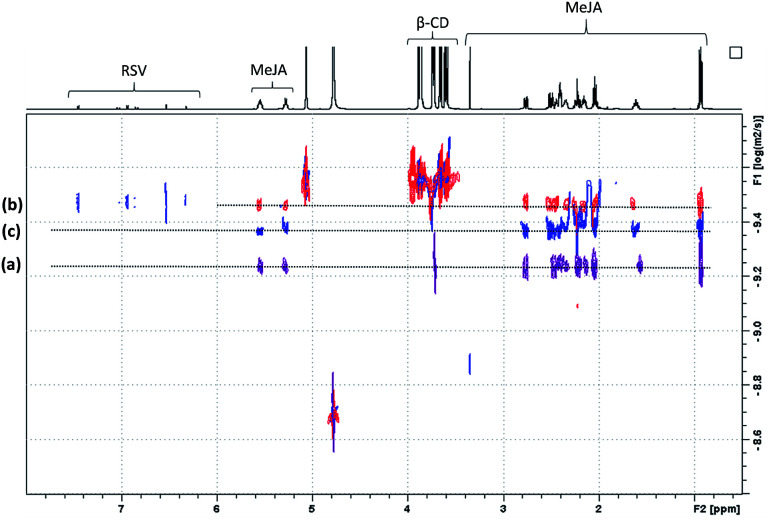
NMR DOSY spectra of free MeJA in D_2_O (a), in the presence of β-CD (b) and β-CD/RSV (c).

In order to confirm the T-ROESY results, DOSY NMR experiments were performed as displayed in Fig. 11. In this figure, a *D* value for MeJA alone is observed at 6.12 × 10^−10^ m^2^ s^−1^ (a), reaching 3.37 × 10^−10^ m^2^ s^−1^ in the presence of β-CD in agreement with the formation of an inclusion complex (b) and finally increasing to 4.3 × 10^−10^ m^2^ s^−1^ when RSV is added to the mixture (c). At the same time, the RSV *D* value is measured at 3.36 × 10^−10^ m^2^ s^−1^, consistent with the formation of a β-CD/RSV inclusion complex in the ternary mixture. It should be pointed out that a *D* value of 3.29 × 10^−10^ m^2^ s^−1^ was previously determined for RSV in the presence of only β-CD in solution. These results can be explained by the co-existence in solution of two competitive binary inclusion complexes, β-CD/RSV and β-CD/MeJA. Similar results were obtained when β-CD was replaced by DIMEB.

### Studies of RSV formulation stability under UV exposure

In order to investigate the benefit of CDs on RSV stability, different formulations (RSV, RSV/MeJA, RSV/CD, RSV/CD/MeJA with 1/1 molar ratio to be in accordance with the whole study) were prepared and their degradation was followed under UV light irradiation. Resveratrol exists in *trans* and *cis* stereoisomeric forms; the *trans* form (RSV) is more photo- and thermostable but in grapevine the *cis*-resveratrol form is also present. As previously reported by Montsko *et al.*,^42^ UV-photoisomeration of *trans*-resveratrol gives rise to the *cis* isomer and to another product: dehydro-resveratrol, derived from oxidation.

The possible structure of this compound was discussed by Rodriguez-Cabo *et al.*^43^ and corresponds to either a tri hydroxylated phenanthrene (2,4,6-trihydroxy-phenantrene) or a trihydroxylated biphenyl acetylene (3,4′,5-trihydroxy-diphenylacetylene), in which the double bond changes into a triple bond. After preparation in water, in order to preserve the integrity of non-covalent complexes, the formulations were exposed (0 to 37 h) to UV irradiation (253.7 nm) and monitored by LC/UV/HRMS.

**Fig. 12:**
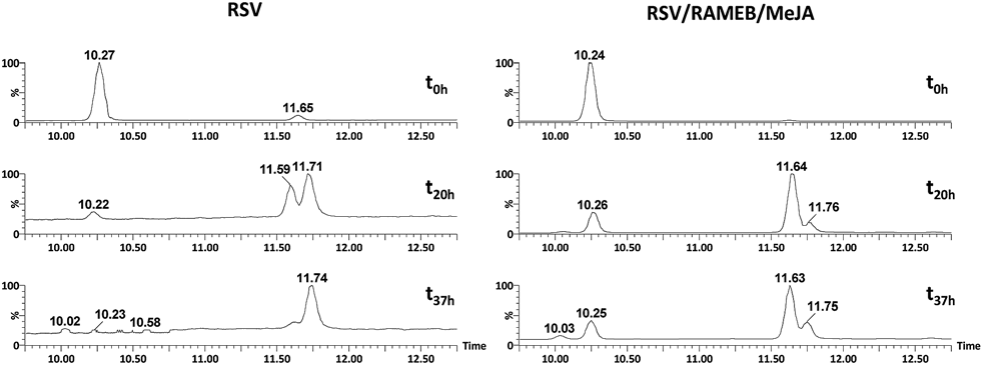
UV traces at 286 and 306 nm of RSV (left) and RSV/RAMEB/MeJA (right) after UV exposure at *t*_0 h_ (top), *t*_20 h_ (middle) and *t*_37 h_ (bottom).

**Fig. 13:**
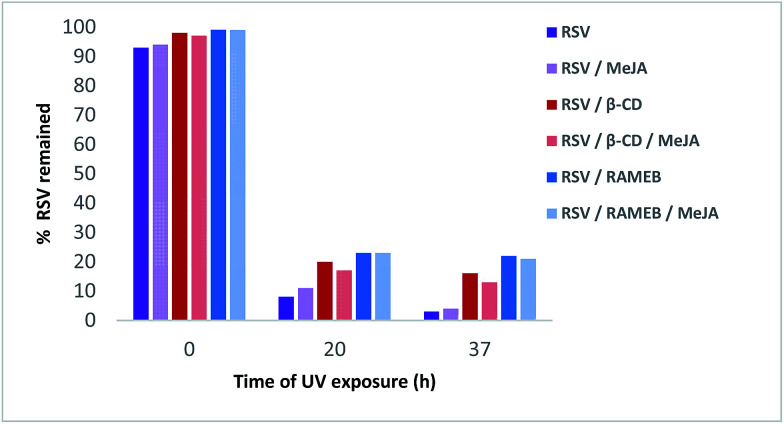
Remaining% of RSV in different formulations under UV light irradiation.

As shown in Fig. 12, RSV (10.27 min) undergoes a severe degradation leading to *cis*-resveratrol (11.59 min) and dehydro-resveratrol (11.74 min). After 37 h, the proportion of the remaining RSV is less than 3% (Fig. 13) and the main compound observed in the UV trace corresponds to the oxidate derivative.

The respective structures were confirmed by ESI-MS/MS of the [M − H]^−^ species at *m*/*z* 227.071 (*trans* and *cis*-resveratrol, C_14_H_11_O_3_) and *m*/*z* 225.055 (dehydro-resveratrol, C_14_H_9_O_3_). The main product ions observed for resveratrol: 185.057 and 143.046 and for dehydro-resveratrol: 180.056 and 155.047 are identical to those described by Rodriguez-Cabo *et al.*^43^

Whereas with the ratio 1/1 of CD *vs.* RSV, the majority of RSV is not complexed, stability is clearly increased for example 7.3-fold with RAMEB. In the presence of MeJA, the degradation process of RSV (Fig. 13) remains the same, with the protective effect of CDs being clearly evidenced whether MeJA is present or not. The best result is obtained for RAMEB where 22% of RSV remains after 37 h exposure and the protective effect is already effective at *t*_0 h_ (Fig. 12 and 13). With a more favorable ratio of CD *vs.* RSV, one can put forth the hypothesis of an increasing stabilization of RSV.

## Experimental

### Materials and methods

#### Materials

*trans*-Resveratrol (RSV), methyl jasmonate (MeJA) and maltoheptaose were purchased from Sigma Aldrich (Saint Quentin Fallavier, France). α-CD, β-CD, γ-CD were purchased from Wacker Chimie (Lyon, France), DIMEB from Cis BIO (Saclay, France), and RAMEB from Roquette (Lestrem, France). For liquid chromatography and mass spectrometry, water, methanol and formic acid (ULC-MS grade) were purchased from Biosolve (Dieuze, France). D_2_O for NMR experiments was purchased from CortecNet (Voisins le Bretonneux, France).

#### Solubility studies

Phase solubility studies were carried out according to the Higuchi and Connors method.^26^ Excess amounts of RSV were added (3–5 mg) to a 5 mL aqueous solution containing different CD concentrations (from 2.5 to 20 mM). The suspensions were maintained at 25 °C for 18 h with stirring. Then, an aliquot was filtered through 0.2 μm cellulose filters from Grace (Templemars, France), 1 : 100 diluted and analyzed by UV (*λ*_max_ = 306 nm) using a JASCO V-630 spectrophotometer (Lisses, France).

#### Stability studies by ESI-HRMS

The stability of RSV-CD and MeJA-CD complexes was studied by high resolution mass spectrometry (HRMS) on a Q-TOF Ultima Global hybrid quadrupole time-of-flight instrument (Waters-Micromass, Manchester, UK), equipped with a pneumatically assisted electrospray (ESI) ionization source (Z-spray) and an additional sprayer for the reference compound (Lock Spray). ESI-HRMS data were recorded in the positive ion mode. The source and desolvation temperatures were 80 and 120 °C, respectively. Nitrogen was used as a drying and nebulizing gas at flow rates of 350 and 50 L h^−1^, respectively. Typically, the capillary voltage was 3 kV and the cone voltage varied from 35 to 195 V. Lock mass corrections, using appropriate cluster ions of an orthophosphoric acid solution (0.1% in H_2_O/CH_3_CN 50/50, v/v), were applied for accurate mass measurements (elemental composition determination). The mass range was 50–3000 Da and spectra were recorded at 3 s per scan in the profile mode at a resolution of 10 000 (FWHM). Data acquisition and processing were performed with MassLynx 4.0 software.

To calculate the relative intensities of each CD complex with RSV or MeJA, the following equation was used:
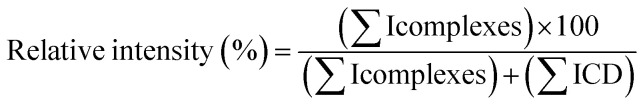


In practice, for each cone voltage value (starting from 35 V and ramping up to 195 V) the relative intensity of ions from CD/RSV and CD/MeJA complexes *vs.* free CDs was measured on MS spectra taking into account all ion species *i.e.* monocharged and multicharged ions.

#### LC/UV/HRMS studies of RSV formulations under UV exposure

Formulations (RSV, RSV/MeJA, CD/RSV, CD/RSV/MeJA) were prepared in water, in order to preserve the non-covalent complexes, using equimolar solutions (4.4 μmol mL^−1^) of each individual compound (RSV, MeJA, CD). After preparation, the different formulations were exposed to UV irradiation at 253.7 nm and analyzed by LC/UV/HRMS at *t*_0 h_, *t*_20 h_ and *t*_37 h_ using the Q-TOF Ultima Global hybrid quadrupole time-of-flight instrument (Waters-Micromass, Manchester, UK), coupled with an Ultra-Fast Liquid Chromatography (UFLC) system (Shimadzu, Champs-sur-Marne, France). Samples were loaded (5 μL) on a Symmetry C8 column (3.5 μm, 100 × 4.6 mm) (Waters, Guyancourt, France) set at 30 °C. The elution was performed using a 1 mL min^−1^ mobile phase gradient programmed from water containing 0.1% formic acid (A) and acetonitrile containing 0.1% formic acid (B) as follows (A/B): 95 : 5 (*t* = 0 min), 5 : 95 (*t* = 30 min), 5 : 95 (*t* = 35 min), 95 : 5 (*t* = 40 min) and 95 : 5 (*t* = 45 min). The effluent was flow-split *via* a peek tee with 1/5 of the flow directed toward the electrospray (ESI) source and the residual 4/5 directed toward the UV detector recording the two RSV specific wavelengths at 286 and 306 nm. LC/ESI-MS data were recorded in the negative ion mode with a capillary voltage of – 2 kV and a cone voltage of – 50 V. The source and desolvation temperatures were kept at 120 and 250 °C, respectively. Nitrogen was used as a drying and nebulizing gas at flow rates of 450 and 100 L h^−1^, respectively. For MS, scanning was performed in the 50–2000 Da range at a scan rate of 2 s per scan. For MS/MS, argon was used as collision gas and the collision energy was set to 25 V, the mass range was 50–300 Da at a scan rate of 0.5 s per scan. As for the ESI-HRMS stability studies, spectra were collected in the profile mode and data acquisition and processing were performed with MassLynx 4.0 software.

#### NMR studies

All NMR experiments were performed at 600.17 MHz using a Bruker Avance III spectrometer equipped with a Z-gradient unit for pulsed-field gradient spectroscopy and with a 5 mm TXI probe. Calibration was performed using the signal of the residual protons of the solvent (HOD) as a reference. Measurements were performed at 300 K with careful temperature regulation. The length of the 90° pulse was approximately 7 μs. 1D NMR data spectra were collected using 16 K data points. 2D experiments were run using 1 K data points and 512 time increments. The phase sensitive (TTPI) sequence was used and processing resulted in a 1 K × 1 K (real–real) matrix. Details of the experimental conditions are given in the figure captions. For diffusion coefficient measurements, 2D ^1^H DOSY NMR experiments were carried out using the Bruker sequence ledbpgp2s. Gradient calibration of the probe was done using the water signal from an H_2_O/D_2_O (90/10) mixture; the gradient value *G* = 4.9 G mm^−1^ was obtained for a water diffusion value *D* at 2.3 × 10^−9^ m^2^ s^−1^ at 25 °C. The strength of the pulsed-field gradient was linearly increased from 2 to 95% in 16 steps. The diffusion time (*Δ*) and the gradient duration (*δ*/2) were set at 80 ms and 1.5 ms, respectively. The longitudinal eddy current delay and the spoil gradient delay were fixed at 5 and 0.2 ms, respectively. Spectral data were processed *via* the dosy2d module from TOPSPIN software (Bruker). Fourier transform was applied in the F2 dimension for every FID obtained from each programmed gradient value and a baseline correction was applied on every one-dimensional spectrum. An automatic search of the intensity decay for each peak was carried out to obtain the decay time proportional to *D*. Diffusion coefficient values obtained were expressed according to the F1 dimension.

MeJA solubility studies were performed using a ^1^H NMR quantitative experiment called ERETIC.^41^ Prior to this, some preliminary DOSY experiments with different concentrations of MeJA were carried out to find the largest quantity of MeJA that could be solubilized in D_2_O without the formation of bigger objects highlighted with changes in diffusion coefficient. Using this method, the appropriate quantity of MeJA to be introduced into the NMR tube to obtain a saturated D_2_O solution of MeJA was found. Then, increasing concentrations of β-CD and DIMEB from 1 to 10 mM were added to saturated MeJA D_2_O solutions to evaluate the solubilizing effect of CDs on MeJA. The ERETIC method consists of first carrying out a ^1^H NMR experiment of a reference component with a known concentration (citric acid 5 mM in D_2_O) and assigning a number of protons to an integrated signal. Then, this reference spectrum is used to calibrate externally and quantify MeJA after integration of its signals in spectra containing MeJA in the presence of CDs. In ERETIC, the same acquisition parameters and the same baseline correction as the external standard are applied to samples to be quantified.

#### ITC studies

ITC experiments were performed with a Microcal ITC200 microcalorimeter (Microcal Inc., Northampton, MA), at 298 K and pH 8 (phosphate buffer). All solutions were thoroughly degassed prior to investigations. A first 0.5 μL injection was followed by 10 injections of 3.6 μL (time interval between two successive injections: 60 s). The cell volume was equal to 202.8 μL. The stirring speed was kept constant at 1000 rpm.

RSV (0.05 mM) and MeJA (0.5 mM) solutions were independently titrated by cyclodextrin solutions (β-CD or RAMEB, 5 mM). Release experiments were also carried out by injecting a CD and RSV solution on buffer (with respective concentrations of 5 and 0.6 mM in the case of β-CD, 5 and 1 mM in the case of RAMEB). Lastly, a competitive release experiment was conducted by injecting the previous solutions (β-CD or RAMEB with RSV) on a MeJA (0.5 mM) solution. All measurements were performed in triplicate.

Prior to data analysis, control experiments were carried out in the same experimental conditions by injecting individual species on buffer, and/or buffer on species and buffer on buffer. The corresponding thermograms were then appropriately subtracted from the complexation experiments. Baseline subtraction and peak integration were performed using Origin software. The resulting experimental heats were then fitted to theoretical models using in-house dedicated software.^44^ For a given CD, the titration, release and competitive release experiments with RSV and MeJA were treated according to a global analysis: a single set of thermodynamic parameters for each complex (formation constant and inclusion enthalpy) was varied during the simultaneous analysis of all experiments.

## Conclusions

The study of binary systems between βCD, RAMEB and RSV on one hand and between βCD, RAMEB and MeJA on the other hand, by different analytical methods, revealed the formation in water of the following 1 : 1 inclusion complexes: βCD/RSV, RAMEB/RSV, βCD/MeJA and RAMEB/MeJA whose association constant values K are presented in Table 6.

**Table 6:** Association constant values *K* obtained in water at 300 K

Host/guest	RSV	MeJA
β-CD	2415 ± 99 M^−1^	2770 ± 232 M^−1^
RAMEB	18 910 ± 930 M^−1^	1280 ± 180 M^−1^

All the experimental data obtained are in agreement and enable the following summary: βCD/RSV ≪ RAMEB/RSV and βCD/MeJA > RAMEB/MeJA but βCD/RSV is similar to βCD/MeJA while RAMEB/RSV ≫ RAMEB/MeJA, leading to a strong increase in solubility and a significant gain in chemical stability of RSV in the presence of RAMEB.

When the three compounds (CD/RSV and MeJA) were together in aqueous solution, no ternary complex was observed by either NMR or ITC but both inclusion complexes (CD/RSV and CD/MeJA) occurred in solution, probably competitively. In the case of βCD and based on the similar K values, both complexes βCD/RSV and βCD/MeJA must co-exist in similar proportions. In the presence of βCD (13 mM), the results of RSV bioproduction by MeJA (0.8 mM) elicitation show a clear improvement from 32 to 274 mg L^−1^. This may be explained, at least in part, by the formation of an inclusion complex between βCD and MeJA in biological medium since its elicitation effect is less in the absence of βCD. Conversely, in the case of RAMEB, while the two inclusion complexes co-exist also in solution, one (RAMEB/RSV) exhibits a constant value nearly 15 times greater than the other (RAMEB/MeJA). At the same concentrations, the MeJA elicitation effect seems lower in the presence of RAMEB since RSV bioproduction drops from 262 to 170 mg L^−1^. With RAMEB, the main benefits reside in the increase in solubility and chemical stability of RSV due to the very strong affinity of the latter for RAMEB and the possibility to use higher concentrations of this CD in the medium of culture. These results have to be considered with the biosynthesis of RSV, as the main phenomenon is the induction of RSV production by MeJA and CDs acting as elicitors through the classical signaling pathways in grapevine cells.^20^ The presence of βCD–MeJA complexes in a higher quantity than RAMEB/MEJA complexes could enhance the solubility of MEJA in the water liquid medium, and then provoked a highest elicitation response. To conclude, it might be interesting to evaluate the effect of a βCD/RAMEB mixture on RSV bioproduction. It may be possible to cumulate the βCD positive effect on the elicitation by MeJA and those of RAMEB on the solubilization and protection of RSV in the culture medium.

## Conflicts of interest

There are no conflicts to declare.

## Supplementary Material

RA-008-C7RA11619E-s001

## References

[cit1] Romero-Pérez A. I., Lamuela-Raventós R. M., Waterhouse A. L., de la Torre-Boronat M. C. (1996). J. Agric. Food Chem..

[cit2] Nour V., Trandafir I., Muntean C. (2012). J. Chromatogr. Sci..

[cit3] Cote C. D., Rasmussen B. A., Duca F. A., Zadeh-Tahmasebi M., Baur J. A., Daljeet M., Breen D. M., Filippi B. M., Lam T. K. T. (2015). Nat. Med..

[cit4] Catalgol B., Batirel S., Tagaand Y., Ozer N. (2012). Front. Pharmacol..

[cit5] Chen H., Deng Q., Ji X., Zhou X., Kelly G., Zhang J. (2016). New J. Chem..

[cit6] Calzarano F., D'Agostino V., Del Carlo M. (2008). Anal. Lett..

[cit7] Donnez D., Jeandet P., Clément C., Courot E. (2009). Trends Biotechnol..

[cit8] Donnez D., Kim K.-H., Antoine S., Conreux A., De Luca V., Jeandet P., Clément C., Courot E. (2011). Process Biochem..

[cit9] Krisa S., Larronde F., Budzinski H., Decendit A., Deffieux G., Merillon J. M. (1999). J. Nat. Prod..

[cit10] Taurino M., Ingrosso I., D'amico L., De Domenico S., Nicoletti I., Corradini D., Santino A., Giovinazzo G. (2015). SpringerPlus.

[cit11] Belhadj A., Telef N., Saigne C., Cluzet S., Barrieu F., Hamdi S., Merillon J. M. (2008). Plant Physiol. Biochem..

[cit12] Lu Z., Chen R., Fu R., Xiong J., Hu Y. (2012). J. Inclusion Phenom. Macrocyclic Chem..

[cit13] Cyclodextrins and their complexes, ed. H. Dodziuk, Wiley-VCH, Weinheim, 2006, p. 489

[cit14] Mathiron D., Marçon F., Dubaele J.-M., Cailleu D., Pilard S., Djedaïni-Pilard F. (2013). J. Pharm. Sci..

[cit15] Casu B., Reggiani M., Sanderson G. R. (1979). Carbohydr. Res..

[cit16] Nivelle L., Hubert J., Courot E., Jeandet P., Aziz A., Nuzillard M., Renault J. H., Clément C., Martiny L., Delmas D., Tarpin M. (2017). Molecules.

[cit17] Jeandet P., Clément C., Tisserant L. P., Crouzet J., Courot E. (2016). C. R. Chim..

[cit18] Lijavetzky D., Almagro L., Belchi-Navarro S., Martinez-Zapater J. M., Bru R., Pedreno M. A. (2008). BMC Res. Notes.

[cit19] Belchí-Navarro S., Almagro L., Lijavetzky D., Bru R., Pedreño M. A. (2012). Plant Cell Rep..

[cit20] Almagro L., Carbonell-Bejerano P., Belchí-Navarro S., Bru R., Martínez-Zapater J. M., Lijavetzky D., Pedreño M. A. (2014). PLoS One.

[cit21] Lucas-Abellán C., Fortea I., López-Nicolás J. M., Núñez-Delicado E. (2007). Food Chem..

[cit22] Bertacche V., Lorenzi N., Nava D., Pini E., Sinico C. (2006). J. Inclusion Phenom. Macrocyclic Chem..

[cit23] Lucas-Abellán C., Fortea M. I., Gabaldón J. A., Núñez-Delicado E. (2008). Food Chem..

[cit24] Trollope L., Cruickshank D. L., Noonan T., Bourne S. A., Sorrenti M., Catenacci L., Caira M. R. (2014). Beilstein J. Org. Chem..

[cit25] Caira M. R., Bourne S. A., Samsodien H., Smith V. J. (2015). Beilstein J. Org. Chem..

[cit26] Higuchi T. and Connors K., Advances in Analytical Chemistry and Instrumentation, Wiley Intersciences, New York, 1965, pp. 117–212

[cit27] Brewster M. E., Loftsson T. (2007). Adv. Drug Delivery Rev..

[cit28] Zhou R., Wang F., Guo Z., Zhao Y. (2012). J. Food Process Eng..

[cit29] Loftsson T., Hreinsdóttir D., Másson M. (2007). J. Inclusion Phenom. Macrocyclic Chem..

[cit30] Ganem B., Li Y. T., Henion J. D. (1991). J. Am. Chem. Soc..

[cit31] Gabelica V., Galic N., De Pauw E. (2002). J. Am. Soc. Mass Spectrom..

[cit32] Lu Z., Chen R., Liu H., Hu Y., Cheng B., Zou G. (2009). J. Inclusion Phenom. Macrocyclic Chem..

[cit33] Ishikawa M., Sueishi Y., Endo N., Oowada S., Shimmei M., Fujii H., Kotake Y. (2011). Int. J. Chem. Kinet..

[cit34] Troche-Pesqueira E., Pérez-Juste I., Navarro-Vásquez A., Cid M. M. (2013). RSC Adv..

[cit35] Sapino S., Carlotti M. E., Caron G., Ugazio E., Cavalli R. (2009). J. Inclusion Phenom. Macrocyclic Chem..

[cit36] López-Nicolás J. M., García-Carmona F. (2010). Food Chem..

[cit37] Venuti V., Cannavà C., Cristiano M. C., Fresta M., Majolino D., Paolino D., Stancanelli R., Tommasini S., Ventura C. A. (2014). Colloids Surf., B.

[cit38] López-Nicolás J. M., García-Carmona F. (2008). Food Chem..

[cit39] Li H., Xu X., Liu M., Sun D., Li L. (2010). Thermochim. Acta.

[cit40] López-Nicolás J. M., Camps M. E., Perez-Sanchez H., García-Carmona F. (2013). J. Agric. Food Chem..

[cit41] Akoka S., Barantin L., Trierweiler M. (1999). Anal. Chem..

[cit42] Montsko G., Pour Nikfardjam M. S., Szabo Z., Boddi K., Lorand T., Ohmacht R., Mark L. (2008). J. Photochem. Photobiol., C.

[cit43] Rodriguez-Cabo T., Rodríguez I., López P., Ramil M., Cela R. (2014). J. Chromatogr. A.

[cit44] Bertaut E., Landy D. (2014). Beilstein J. Org. Chem..

