# Supplementary material for: Physico-chemical studies of resveratrol, methyl-jasmonate and cyclodextrin interactions: an approach to resveratrol bioproduction optimization
Source: RSC Adv. 2018 Jan 4;8(3):1528–38. doi: 10.1039/c7ra11619e (PMC9128506; doi:10.1039/c7ra11619e)
Supplement: RA-008-C7RA11619E-s001 [file RA-008-C7RA11619E-s001.pdf]

Electronic Supplementary Material (ESI) for RSC Advances.
This journal is © The Royal Society of Chemistry 2017

Physico chemical studies of resveratrol, methyl-jasmonate and cyclodextrins interactions: an approach to resveratrol bioproduction optimization

E. Oliva[a,b,e], D. Mathiron[b,e], E. Bertaut[c,e], D. Landy[c,e], D. Cailleu[b,e], S. Pilard[b,e], C. Clément[d,e], E. Courot[d,e], V. Bonnet[a,e] and F. Djedaïni-Pilard[a,e*]

[a.] *Laboratoire de Glycochimie des Antimicrobiens et des Agroressources UMR 7378 CNRS, Université de Picardie Jules Verne, 33 rue Saint-Leu, 80039 Amiens, France.*

[b.] *Plateforme-analytique, Université de Picardie Jules Verne, 33 rue Saint-Leu, 80039 Amiens, France.*

[c.] *Unité de Chimie Environnementale et Interactions sur le Vivant (UCEIV, EA 4492), ULCO, F-59140 Dunkerque, France.*

[d.] *URVVC EA 4707, Université de Reims Champagne-Ardenne, Moulin de la Housse, 51687 Reims, France.*

[e.] *SFR Condorcet "Agrosciences Environnement et Développement Durable" FR CNRS 3417, UFR Sciences Exactes et Naturelles, BP 1039 Moulin de la Housse - Bâtiment 18 - 51687 Reims Cedex 02, France.*

Supplementary Information

Figure S1: ESI (+) MS Spectra corresponding to RSV with maltoheptaose (a) and RSV with β-CD (b) in the same experimental conditions.

Figure S2: dedicated Job plots of (a) β-CD (up)/resveratrol (down) and (b) DIMEB (up)/resveratrol (down)

Figure S3: Plot of the relative intensity of each complex between CD and MeJA versus cone voltage.

Figure S4: Fit between experimental RSV solubility data (β-CD ■,and RAMEB ×) and theoretical values predicted by formation constants obtained by ITC (red line and blue line, respectively).

Figure S5: Fit between experimental MEJA solubility data (β-CD ■,and RAMEB ×) and theoretical values predicted by formation constants obtained by ITC (red line and blue line, respectively).

Table SI : Association constant values, K (M^{-1}) calculated from solubility data

Figure S6: Enlarged contour plot of a T-ROESY experiment (spin lock: 300 ms, 22 dB) performed at 600 MHz on a β-CD/RSV/MeJA mixture (4.6 mM of β-CD with RSV and MeJA at saturation in D_2O). Rectangles represent the absence of a correlation between RSV and MeJA and ovals the presence of correlations between β-CD/ RSV on one hand and β-CD/MeJA on the other hand

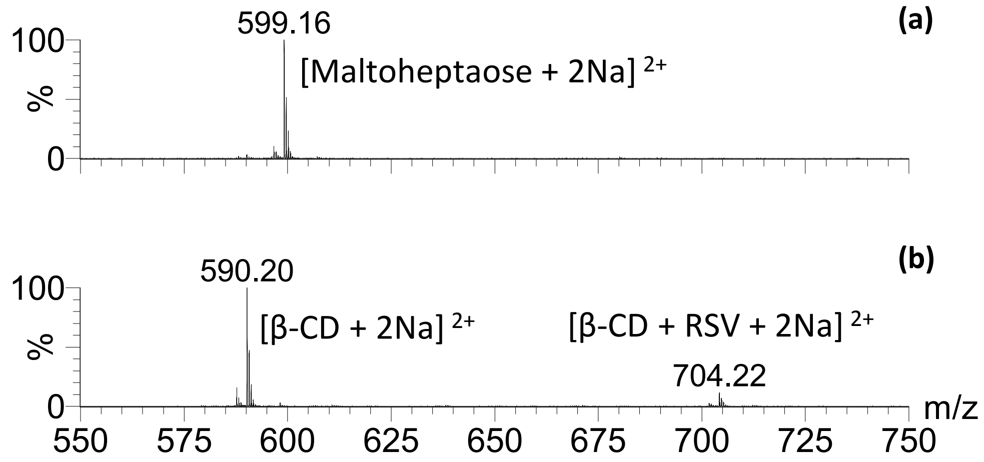

Figure S1: ESI (+) MS Spectra corresponding to RSV with maltoheptaose (a) and RSV with β-CD (b) in the same experimental conditions.

a

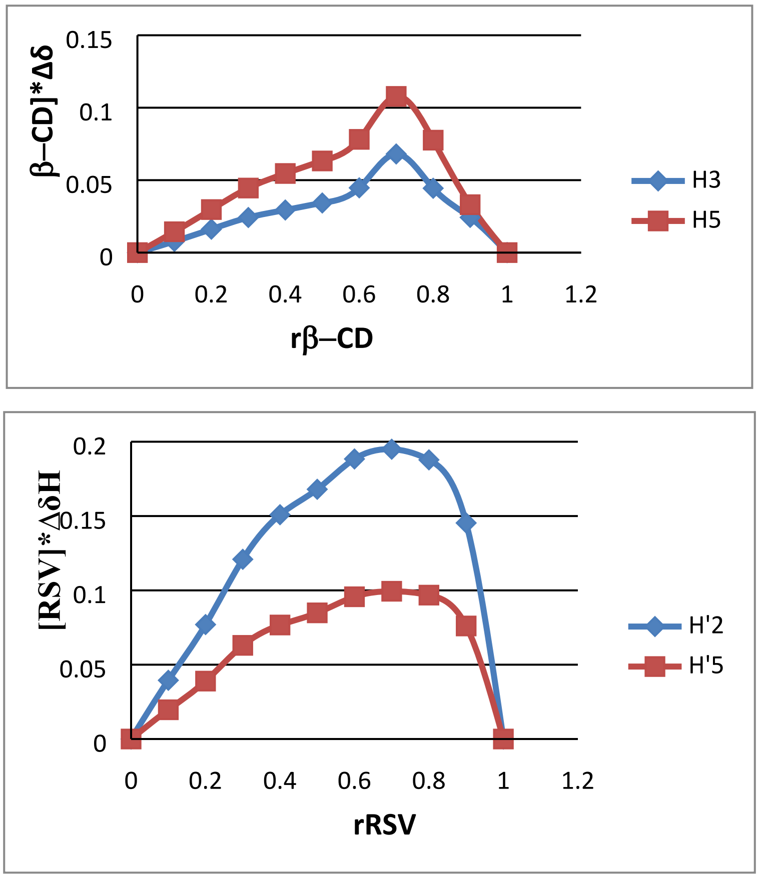

b

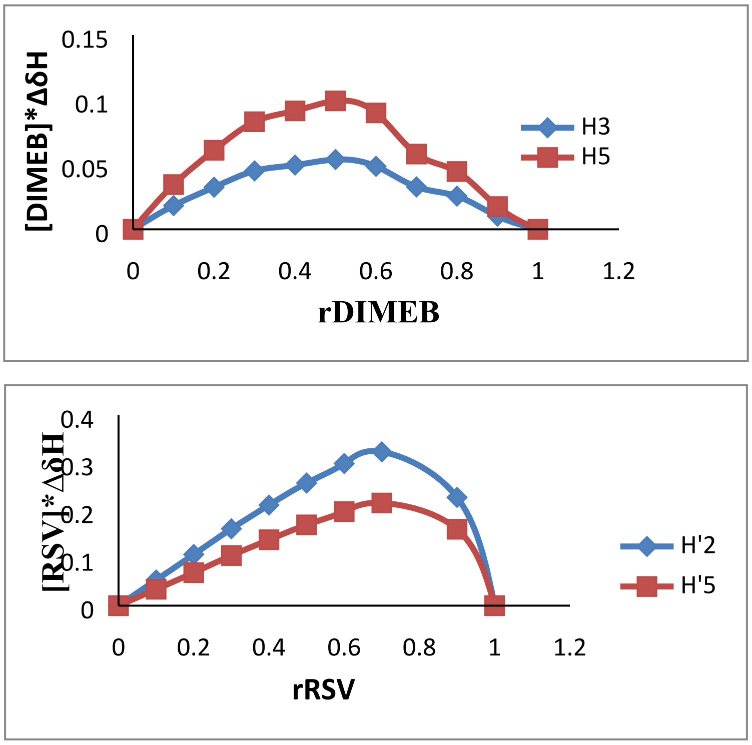

Figure S2: dedicated Job plots of (a) β-CD (up)/resveratrol (down) and (b) DIMEB (up)/resveratrol (down)

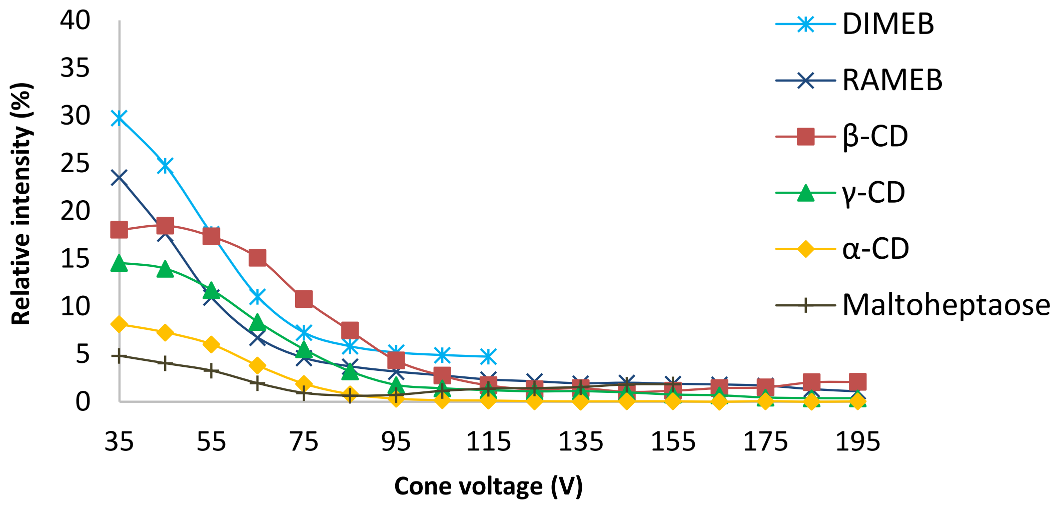

Figure S3: Plot of the relative intensity of each complex between CD and MeJA versus cone voltage.

The solubility of a guest G in the presence of CD (leading to a CD-G complex with a formation constant K), can be calculated as follows:

$$[G]_T = [G] + [CD\text{-}G] \quad (1)$$

$$[CD]_T = [CD] + [CD\text{-}G] \quad (2)$$

$$K = [CD\text{-}G] / ([CD]*[G]) \quad (3)$$

Combining equation (3) with equation (2):

$$[CD\text{-}G] = K * ([CD]_T - [CD\text{-}G]) * [G] \quad (4)$$

In phase solubility experiments, [G] is equal to the intrinsic G solubility S_0, thus:

$$[CD\text{-}G] = (K*[CD]_T*S_0) / (1+K*S_0) \quad (5)$$

As a result, the total guest concentration (ie. its solubility in the presence of CD) is equal to:

$$[G]_T = [G] + [CD\text{-}G] = S_0 + (K*[CD]_T*S_0) / (1+K*S_0) \quad (6)$$

Accordingly, formation constants obtained by ITC were used to simulate the RSV and MEJA solubility in the presence of β-CD and RAMEB. Least square fitting between experimental and theoretical data were then realized (Figure S4 and S5, for RSV and MEJA, respectively), with a floating value for the intrinsic guest solubility.

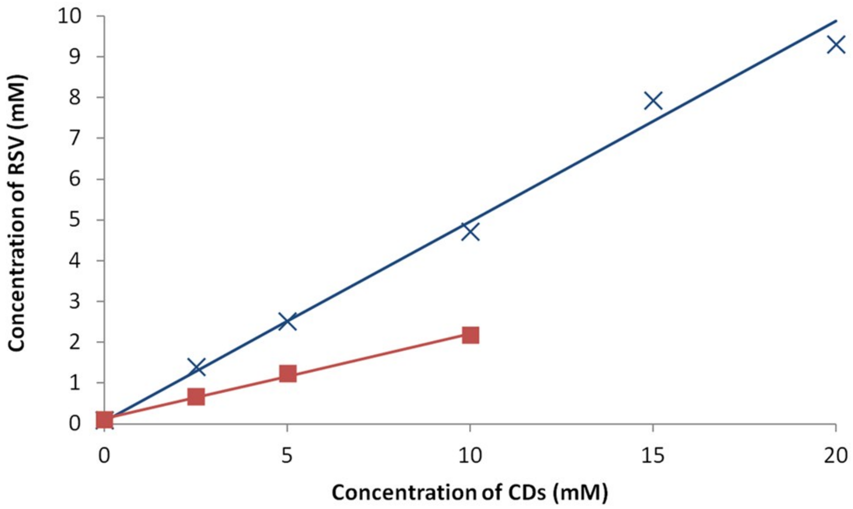

Figure S4: Fit between experimental RSV solubility data (β-CD ■ and RAMEB ×) and theoretical values predicted by formation constants obtained by ITC (red line and blue line, respectively).

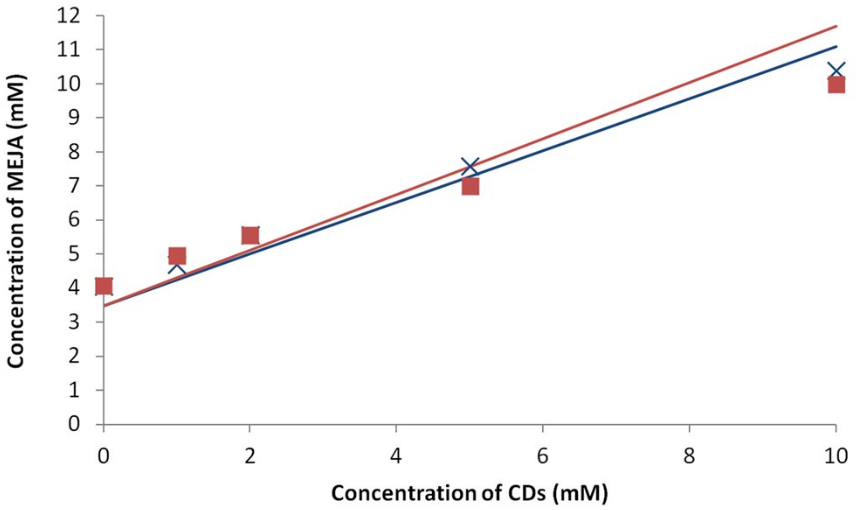

Figure S5: Fit between experimental MeJA solubility data (β-CD ■ and RAMEB ×) and theoretical values predicted by formation constants obtained by ITC (red line and blue line, respectively).

	RSV	MeJA
β-CD	3 053	481
RAMEB	10 700	570

Table SI : Association constant values, K (M^{-1}) calculated from solubility data

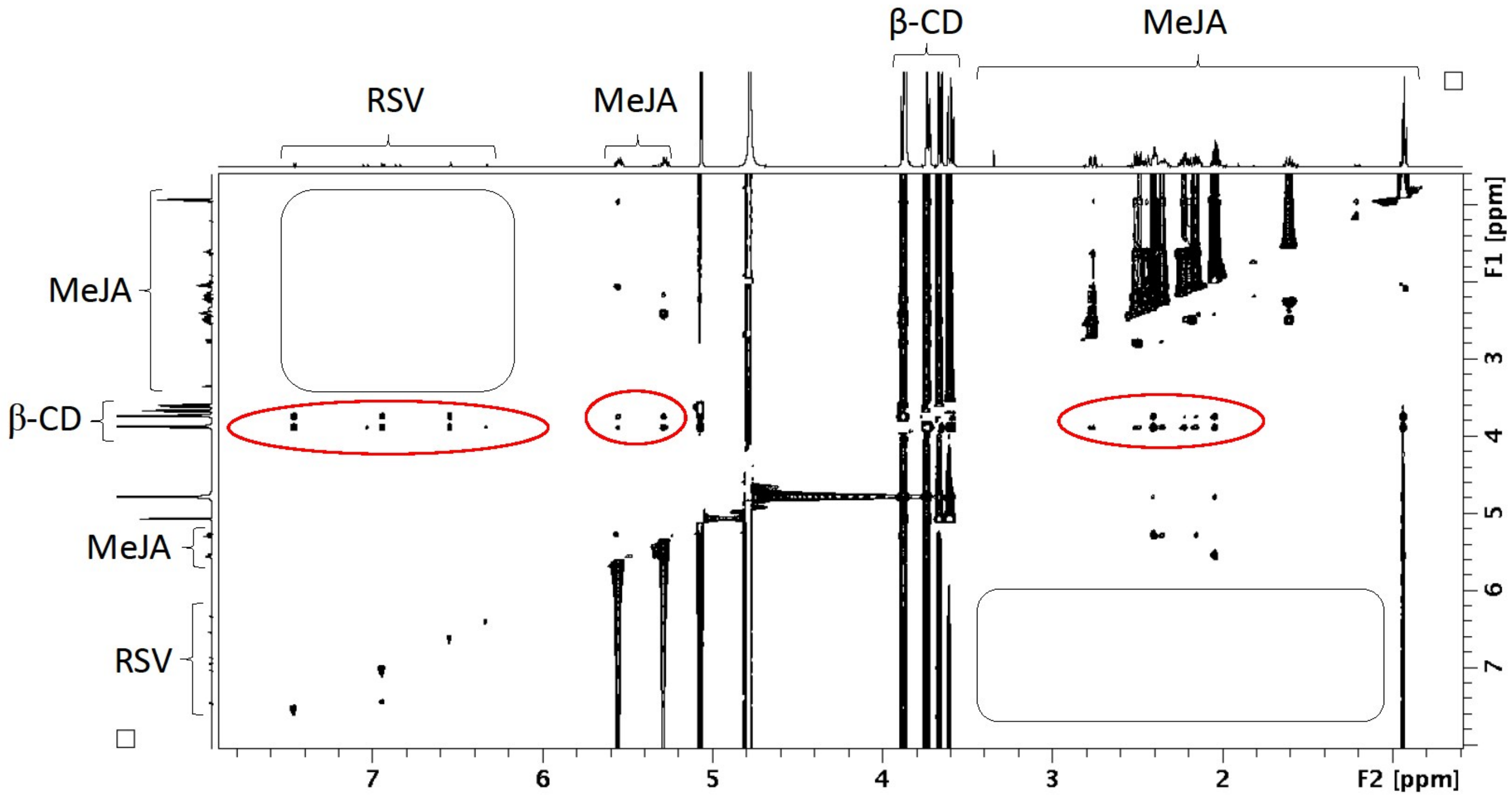

Figure S6: Enlarged contour plot of a T-ROESY experiment (spin lock: 300 ms, 22 dB) performed at 600 MHz on a β-CD/RSV/MeJA mixture (4.6 mM of β-CD with RSV and MeJA at saturation in D_2O). Rectangles represent the absence of a correlation between RSV and MeJA and ovals the presence of correlations between β-CD/ RSV on one hand and β-CD/MeJA on the other hand
